# The vascular endothelium: the cornerstone of organ dysfunction in severe SARS-CoV-2 infection

**DOI:** 10.1186/s13054-020-03062-7

**Published:** 2020-06-16

**Authors:** Stéphanie Pons, Sofiane Fodil, Elie Azoulay, Lara Zafrani

**Affiliations:** 1 INSERM U976, Human Immunology, Pathophysiology and Immunotherapy, Saint-Louis Teaching Hospital, Paris University, Paris, France; 2 Anesthesia and Critical Care Department, Avicenne Teaching Hospital, Assistance Publique des Hôpitaux de Paris, Bobigny, France; 3 Department of Medical Intensive Care Unit, Saint-Louis Teaching Hospital, Assistance Publique des Hôpitaux de Paris, 1, Avenue Claude Vellefaux, 75010 Paris, France

**Keywords:** SARS-CoV-2, COVID-19, Endothelial cells, Endothelial dysfunction, Cytokines, Thrombosis

## Abstract

In severe SARS-CoV-2 infections, emerging data including recent histopathological studies have emphasized the crucial role of endothelial cells (ECs) in vascular dysfunction, immunothrombosis, and inflammation.

Histopathological studies have evidenced direct viral infection of ECs, endotheliitis with diffuse endothelial inflammation, and micro- and macrovascular thrombosis both in the venous and arterial circulations. Venous thrombotic events, particularly pulmonary embolism, with elevated D-dimer and coagulation activation are highly prevalent in COVID-19 patients. The pro-inflammatory cytokine storm, with elevated levels of interleukin-6 (IL-6), IL-2 receptor, and tumor necrosis factor-α, could also participate in endothelial dysfunction and leukocyte recruitment in the microvasculature. COVID-19-induced endotheliitis may explain the systemic impaired microcirculatory function in different organs in COVID-19 patients. Ongoing trials directly and indirectly target COVID-19-related endothelial dysfunctions: i.e., a virus-cell entry using recombinant angiotensin-converting enzyme 2 (ACE2) and transmembrane protease serine 2 (TMPRSS-2) blockade, coagulation activation, and immunomodulatory therapies, such as anti-IL-6 strategies. Studies focusing on endothelial dysfunction in COVID-19 patients are warranted as to decipher their precise role in severe SARS-CoV-2 infection and organ dysfunction and to identify targets for further interventions.

## Background

Since December 2019, a novel betacoronavirus named SARS-CoV-2 (severe acute respiratory syndrome coronavirus-2) has caused a global outbreak of respiratory illness described as COVID-19. SARS-CoV 2 infection induces a viral pneumonia that leads to acute respiratory failure in up to 20% of symptomatic patients [1, 2]. At early stages of the pandemic, little attention has been paid to endothelial dysfunction in severe SARS-CoV-2 infection. Yet, endothelial cells (ECs) have a crucial role in several physiologic processes. They control blood rheology, vasomotor tone regulation, osmotic balance, and vascular barrier function [3, 4]. The endothelium has also a key role in setting up the innate immune response in a wide array of critical care conditions, such as sepsis, but it exhibits intrinsic properties involved in the activation of adaptive immunity [5–7]. ECs represent an important target for infection of most human viruses, enhancing immune response, inducing increased tissue permeability, inflammation, and contributing to the severity of the viral disease [8]. Indeed, ECs in humans basally express both class I and class II MHC molecules [9]. Thus, they are able to process antigens (Ag) and act as antigen-presenting cells. ECs cannot activate naïve lymphocytes but can mediate Ag-specific stimulation of Ag effector or memory CD4 and CD8 lymphocytes [10–12]. Moreover, endothelial dysfunction is known to be highly involved in organ dysfunction during viral infections, as it induces a pro-coagulant state, microvascular leak, and organ ischemia [13]. In SARS-CoV-2 infections, emerging data including recent histopathological studies have highlighted the crucial role of ECs in vascular dysfunction, inflammation, and (immuno) thrombosis [14, 15].

## Histological evidence of endothelial dysfunction during SARS-CoV-2 infection

In vitro, SARS-CoV-2 is able to directly infect engineered human blood vessel organoids [16]. In three patients infected with SARS-CoV-2, Varga et al. described endothelial cell involvement in different organs, including the kidney, lung, heart, and liver. They found evidence of viral inclusion structures in ECs, as well as endothelial inflammation with the recruitment of neutrophils and mononuclear cells. Indeed, by electron microscopy, they identified viral inclusion in endothelial cells from a transplanted kidney. In another critically ill patient with multi-organ failure, post-mortem histology revealed lymphocytic endotheliitis in the same organs. In another COVID-19 patient with mesenteric ischemia, histology of the small intestine resection disclosed prominent endotheliitis of the submucosal vessels with evidence of direct viral infection of the ECs and diffuse endothelial inflammation with mononuclear cell infiltrate. Authors suggest that COVID-19-induced endotheliitis may explain the systemic impaired microcirculatory function in different organs in COVID-19 patients [14]. Severe COVID-19 is associated with cytokine secretion and immune cell recruitment that undoubtedly result in EC activation [17]. Given the fundamental role of ECs in maintaining homeostasis, vascular permeability, and blood rheology, EC dysfunction may actively participate in thrombo-inflammatory processes that ultimately result in COVID-19 vasculopathy, ventilation-perfusion mismatch, and a clinical phenotype of refractory ARDS [18].

In a post-mortem histopathological analysis of 26 patients who died because of SARS-CoV-2 infection, Su et al. found evidence of coronavirus particles in the tubular epithelium and podocytes but not in renal ECs. However, they found endothelial cell swelling with foamy degeneration in five patients. Among them, three patients had a few areas of segmental fibrin thrombus in glomerular capillary loops associated with a severe endothelial injury. Whether these findings are indicative of specific endothelial injury due to SARS-CoV-2 invasion or should they reflect the severity of underlying conditions such as hypertension or diabetes that are present in more than half of severe COVID-19 patients is unclear [19]. In post-mortem lung biopsies performed in 6 patients who died from SARS-CoV-2 infection, Copin et al. showed that vascular injury was also a prominent feature, demonstrated by endothelial injury with cytoplasmic vacuolization and cell detachment in small to medium-sized pulmonary arteries [20].

## Entry of SARS-CoV-2 into endothelial cells

Angiotensin-converting enzyme 2 (ACE2) is a homolog of ACE that converts angiotensin II to angiotensin 1–7, which alleviates renin-angiotensin system-related vasoconstriction. SARS-CoV-2 binds with ACE2 on the cell membrane of the host cells. ACE2 has been found in arterial and venous endothelial cells in various human tissues, including the oral and nasal mucosa, lung, small intestine, colon, skin, lymph nodes, thymus, bone marrow, spleen, kidney, and brain [21]. ACE2 presence on the endothelia of various organs does not necessarily imply that SARS-CoV-2 invades all organs. Indeed, evidence of virus invasion has not been found in all human tissues even though ACE2 receptors are ubiquitous. Cell entry of coronavirus, which mainly occurs through endocytosis, depends also on the binding of the viral spike (S) proteins to cellular receptors and on S protein priming by host cell proteases [22]. Therefore, cell invasion depends on both ACE2 expression and the availability of the protease transmembrane protease serine 2 (TMPRSS-2), or other proteases, to cleave the viral spike [23]. TMPRSS-2 has been also previously shown to be expressed in human endothelial cells, but its expression may vary among microvascular and macrovascular beds and across organs [24].

## Cytokines and endothelial activation during SARS-CoV-2 infection

ECs display important immunologic functions. They participate in the regulation of local and systemic inflammatory and immune reactions [17, 25]. Thus, ECs can interact with complement, chemokines, or humoral components and generate or respond to cytokines [3]. Cytokines and chemokines have long been thought to play an important role in immunity and immunopathology during virus infections, such as influenza or coronavirus infections [26, 27].

Cytokine release syndrome (CRS) is a systemic inflammatory response, which can be caused by an infection, some drugs, or cancers and is characterized by a major increase in the level of a large number of pro-inflammatory cytokines. CRS was found to be the major cause of morbidity in patients infected with SARS and Middle East respiratory syndrome coronavirus (MERS-CoV) [27]. In patients diagnosed with severe COVID-19, increased levels of pro-inflammatory cytokines, in particular the soluble interleukin 2 receptor (IL-2R), interleukin-6 (IL-6), and tumor necrosis factor-α (TNF-α), have been observed [28]. However, as noticed by Leisman et al. [29], in COVID-19 patients, plasmatic concentrations of cytokines, especially IL-6, are much lower than in typical hyper-inflammatory ARDS or in CRS and should be distinguished from them [30, 31].

IL-6 is a major highly inducible pro-inflammatory cytokine secreted by several different cell types including monocytes, lymphocytes, fibroblasts, and ECs [32, 33]. The main activators of IL-6 expression are interleukin-1β (IL-1β) and TNF-α, viral infection, and angiotensin II [34–36]. IL-6 plays a major role in EC activation during the early phase of inflammation. Thus, IL-6 induces an increased vascular permeability, the secretion of pro-inflammatory cytokine/chemokines by ECs (IL-6, IL-8, and monocyte chemoattractant protein-1 (MCP-1)), and the activation of C5a complement [37, 38]. In COVID-19 patients, levels of IL-6 seem directly correlated with the severity of the disease [28, 39, 40]. These findings were confirmed in a recent meta-analysis of observational Chinese studies [28, 41–46]. Moreover, levels of IL-6 were significantly associated with mortality. In this study, the authors suggest that IL-6 dosage should be performed to all patients diagnosed with COVID-19 and that the existing scoring system should be customized using IL-6 concentrations [41].

Another important cytokine with increased serum levels observed in COVID-19 is the soluble IL-2R [28, 47]. As for IL-6, levels of soluble IL-2R seem to be correlated with the severity of the disease [47, 48]. Interleukin-2 is mostly secreted by activated T-helper lymphocytes and exerts both stimulatory and regulatory immune functions [49–51]. IL-2 mediates its activity by binding to IL-2R. A lot of different immune cells are expressing the IL-2R at their surface: activated T lymphocytes, regulatory T cells, activated B cells, monocytes, and natural killer cells [52, 53]. The soluble form of the IL-2R seems to be produced by proteolytic cleavage of IL-2Rα, and the release of soluble IL-2R into the circulation is proportional to its membrane expression [54, 55]. Serum levels of soluble IL-2R reflect the immune cell activation in various inflammatory or malignant diseases [56, 57]. It has been also demonstrated that endothelial pulmonary cells express IL-2R on their surface and that IL-2 could bind to endothelial cells and induce a pulmonary-edema in response to this binding [58]. Thus, IL-2R increased serum levels could be induced by the endothelial cells IL-2R expression and IL-2 response might be implicated in the pathophysiology of COVID-19 by a direct action on the endothelium.

Finally, pro-inflammatory cytokines, in particular, IL-1β, IL-6, and TNFα, which are elevated in patients with COVID-19 induce the loss of the normal antithrombotic and anti-inflammatory functions of endothelial cells, leading to coagulation dysregulation, complement and platelet activation, and leukocyte recruitment in the microvasculature [17].

## Thrombotic events in COVID-19 are associated with endothelial cell dysfunction

A remarkable characteristic of patients suffering from severe COVID-19 is the high prevalence of acute thrombotic events. Indeed, several authors have reported acute thrombosis, mainly venous thrombosis and pulmonary embolism in COVID-19 patients. In a cohort of 150 critically ill patients, Helms et al. reported a prevalence of 16.7% of pulmonary embolism and only 2% of deep vein thrombosis [15]. Lodigiani et al. reported that the majority of thrombotic complications were venous and primarily represented by isolated pulmonary embolism [59]. Last, Klok et al. reported a 31% prevalence of thrombotic events in a cohort of 184 critically ill patients [60].

Patients without macrovascular thrombosis, venous or arterial, present with widespread microvascular thrombosis. Histological data have highlighted the role of capillary thrombosis in the pathogenesis of organ dysfunction during SARS-CoV-2 infection. Segmental fibrin thrombi have been found in glomerular capillary loops [19], small fibrinous thrombi in small pulmonary arterioles in areas of both damaged and more preserved lung parenchyma [61], small fibrinous thrombi in the superficial dermal vessels [61], thrombosis of intra-septal microvessels [2], small-sized arterioles with complete luminal thrombosis and occlusive thrombosis of intraseptal capillaries, and medium-sized arteries with complete luminal thrombosis [2].

This high prevalence of pulmonary embolism without deep vein thrombosis, widespread microthrombosis, and fibrin diffuse deposits [62] could be in favor of a dysregulation of coagulation homeostasis initially in the lung that can spread across the body [2]. However, in a recent cohort of 12 consecutive COVID-19-positive deaths, an autopsy revealed deep venous thrombosis in seven of 12 patients (58%) and pulmonary embolism was the direct cause of death in four patients. Moreover, in six of the nine men included in the study, fresh thrombosis was also present in the prostatic venous plexus [63].

Several studies reported coagulation activation, especially in critically ill patients with SARS-CoV-2 infection. Tang et al. reported that among 183 patients, the non-survivors had significantly higher D-dimer and fibrin degradation product (FDP) levels, longer prothrombin time, and activated partial thromboplastin time compared to survivors [64]. Helms et al. found that most patients (> 95%) had elevated D-dimer and fibrinogen levels, but none of them had a positive International Society on Thrombosis and Haemostasis (ISTH) disseminated intravascular coagulation (DIC) score. Moreover, the sepsis-induced coagulopathy score, which detects patients at risk of developing DIC, was positive in only 14.7% of patients. Interestingly, the von Willebrand factor (vWF) activity and vWF antigen (vWF Ag) were considerably increased, as well as factor VIII, indicating inflammation-mediated endothelial activated pro-coagulant state [15]. The main fibrinolytic inhibitor described in the pathogenesis of ARDS is plasminogen activator inhibitor 1 (PAI-1), which is known to be elevated in severe acute respiratory syndrome coronavirus (SARS-CoV) indicating a hypofibrinolytic state associated with the pro-coagulant state [65]. Moreover, direct viral invasion of ECs or indirect activation mediated by complement [66] could be responsible for EC dysfunction and exocytosis of unusually large vWF multimers, as well as platelet activation, leading to microthrombogenesis [67]. Magro et al. described that complement deposition in the lungs of five COVID-19 patients was associated with microvascular injury and thrombosis [68]. The association of the clinical phenotype of COVID-19 respiratory failure, the dysregulated coagulation system with a hypercoagulable state, and elevated endothelial surrogate markers suggests a crucial role played by endothelial damage and inflammation during severe SARS-CoV-2 infection [67].

## The endothelium as a therapeutic target in severe COVID-19 patients

**Fig. 1:**
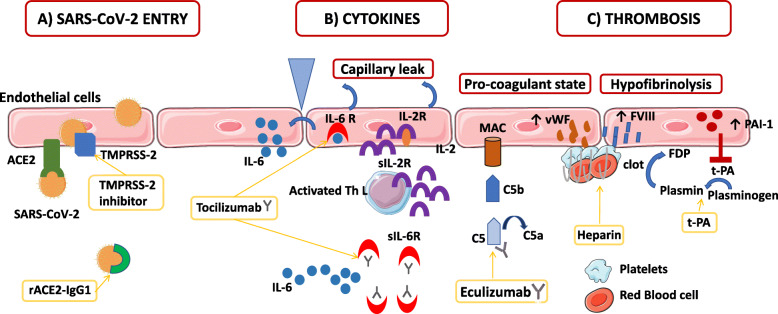
The role of endothelial cells in SARS-CoV-2 infection and treatment. **a** Severe acute respiratory syndrome coronavirus 2 (SARS-CoV-2) binds with Angiotensin- converting enzyme 2 (ACE2) on the cell membrane of the host cells. Cell invasion also depends on the presence of the protease Transmembrane protease serine 2 (TMPRSS-2) that is able to cleave the viral spike. The recombinant protein of human ACE2 fused with the Fc region of the human immunoglobulin IgG1 (rACE2-IgG1) binds with high affinity to the receptor-binding domain of SARS-CoV-2. Moreover, a protease TMPRSS2 inhibitor is efficient to block SARS-CoV-2 entry into the endothelial cells (ECs). **b** In patients diagnosed with severe COVID-19, increased levels of pro-inflammatory cytokines, in particular, the soluble interleukin 2-receptor (IL-2R) and interleukin-6 (IL-6) have been observed. ECs express both IL-6 receptor (IL-6R) and IL-2R on their surface. Soluble IL-2R (sIL-2R) is mostly secreted by activated T helper lymphocytes, but might be also secreted by ECs. Binding of IL-6 and IL-2 on their receptors induces a capillary leak. Moreover, IL-6 signaling induces the secretion by ECs of more IL-6 and other cytokines. Tocilizumab, a humanized anti-IL-6 receptor (IL-6R) antibody that inhibits signal transduction by binding sIL-6R and membrane-bound IL-6R, has emerged as a potential immunomodulatory treatment in COVID-19 patients. **c** During SARS-CoV-2 infection, endothelial dysfunction and microthrombi formation may be secondary to complement activation and membrane attack complexes deposits on ECs. Eculizumab, a human monoclonal antibody designed to bind to the complement protein C5 with high affinity prevents the generation of the terminal membrane attack complex. EC activation also induces a pro-coagulant state by increasing their production of von Willebrand factor (vWF) and factor VIII (FVIII), which participate in clot formation. Heparin from prophylactic to therapeutic doses has been used in COVID-19 patients to inhibit clot formation. Moreover, during SARS-CoV-2 infection, ECs increase their production of plasminogen activator inhibitor 1 (PAI-1), which inhibits the conversion of plasminogen to plasmin and the degradation of clots inducing a hypofibrinolytic state. Tissue plasminogen activator (t-PA) has been tested in COVID-19 patients for its fibrinolytic effect. ACE2: angiotensin-converting enzyme 2; C5: complement C5; FVIII: factor VIII; FDP: fibrin degradation product; IL-2: interleukin-2; IL-6: interleukin-6; IL-2R: interleukin-2 receptor; IL-6-R: interleukin-6 receptor; MAC: membrane attack complex; PAI 1: plasminogen activator inhibitor 1; rACE2-IgG1: recombinant angiotensin-converting enzyme 2 immunoglobulin G1; SARS-Cov-2: severe acute respiratory syndrome coronavirus 2; sIL-2R: soluble interleukin 2 receptor, sIL-6R: soluble interleukin 6 receptor; Th L: T helper lymphocyte; TMPRSS-2: transmembrane protease serine 2 t-PA: tissue plasminogen activator; vWF: von Willebrand factor

To date, COVID-19 patients’ management is only limited to symptomatic or palliative treatments. Ongoing trials directly and indirectly target COVID-19-related endothelial dysfunctions: i.e., a virus-cell entry using recombinant ACE2 and TMPRSS-2 blockade, immunomodulatory therapies such as anti-IL-6 strategies, complement blockade, and coagulation activation (Fig. 1).

## SARS-CoV-2 entry in endothelial cells

The SARS-CoV-2 virus uses ACE2 and TMPRSS2 to infect cells. Thus, the TMPRSS2 blockade has been proposed as a potential treatment. A protease TMPRSS2 inhibitor, already approved for clinical use, has been tested in vitro and is efficient to block SARS-CoV-2 entry into the cells [23]. Recombinant ACE2 (rACE2) is also a molecule of interest in the treatment of COVID-19. Indeed, rACE2 was shown to have therapeutic potential for the SARS-CoV [69]. However, rACE2 exhibits a fast clearance rate, with a half-life of only hours reported by pharmacokinetic studies. Thus, Lei et al. tested the recombinant protein of the extracellular domain of human ACE2 fused with the Fc region of the human immunoglobulin IgG1. This protein has better pharmacological properties and binds with high affinity to the receptor-binding domain of SARS-CoV and SARS-CoV-2. This recent study supports further investigation of ACE2-Ig for diagnosis, prophylaxis, and treatment of SARS-CoV-2 [70]. Some authors have also suggested that renin-angiotensin aldosterone system inhibitors could be a potential treatment of COVID-19, because of the RAAS activation and the key interaction between SARS-CoV-2 and ACE2 (Clinical Trial NCT04311177). However, the balance between circulating ACE2 and membrane-bound ACE2 receptor may be crucial to prevent SARS-CoV-2 entry into target cells which depends on membrane-bound ACE2 receptors [71].

## Immunomodulatory treatments

Corticosteroids are stress-hormones with strong anti-inflammatory activities. They influence the function of various subtypes of immune cells including T cells, dendritic cells, macrophages, and B cells and endothelial cells [72, 73]. Based on previous studies on the use of corticosteroids in SARS, H1N1, and other viral pneumonia, the routine use of glucocorticoids in COVID-19 is not supported by the World Health Organization [74, 75]. Surviving sepsis guidelines recommend to use corticosteroids only for patients in whom adequate fluids, and vasopressor therapy do not restore hemodynamic stability [76]. A recent observational Chinese study of 31 severe COVID-19 patients including 11 treated by corticosteroids found no association between therapy and outcomes in patients without ARDS [77]. However, in a cohort observational study including 201 patients in China, treatment with methylprednisone was beneficial to patients with ARDS [78]. Prospective-controlled randomized studies are ongoing to better determine the place of glucocorticoids in the management of COVID-19 patients (Clinical Trials NCT04344288, NCT04381936, NCT04344730, NCT04348305, and NCT04343729).

Tocilizumab, a humanized anti-IL-6 receptor (IL-6R) antibody that inhibits signal transduction by binding soluble IL-6R and membrane-bound IL-6R, has emerged as a potential immunomodulatory treatment in COVID-19 patients [79]. Two prospective studies without a control arm included respectively 20 and 63 patients with severe or critical COVID-19. Both studies found clinical and biological improvements in patients treated with tocilizumab without obvious adverse reactions [80, 81]. The results from a multicenter randomized clinical trial designed to determine the efficacy and tolerance of tocilizumab in patients with moderate, severe pneumonia, or critical pneumonia associated with COVID-19 is should be disclosed in a close future (Clinical Trial NCT04331808).

## Endothelial dysfunction and pro-thrombotic state

As reviewed in paragraph 3, COVID-19 is frequently associated with thrombotic complications, both in the venous and arterial circulations. Endothelial dysfunction induced by SARS-CoV-2 infection results in a pro-thrombotic state leading to occlusion and microthrombi formation in COVID-19 patients, encouraging the use of prophylactic or even therapeutic anti-coagulation therapies. First, all hospitalized COVID-19 patients should have coagulation tests performed on admission, as it can provide useful prognosis information and help guide the anticoagulant therapy [64, 82, 83]. Tang et al. demonstrated that the use of prophylactic anticoagulant therapy was associated with a decreased mortality in COVID-19 patients [84]. In various national or international guidelines, it has been admitted that all confirmed or suspected COVID-19 patients admitted to the hospital should be treated with venous thrombosis event prophylaxis, in the absence of contra-indication [83, 85]. The dose of anticoagulation for prophylaxis has also been increased by many teams to “intermediate intensity” doses such as 0.5 mg/kg twice a day of enoxaparin, using a risk-adapted strategy based on levels of D-dimer, fibrinogen, ICU location, obesity, or other factors associated with increased risk [85, 86]. The concept of using full-dose anticoagulation in COVID-19 patients for preventing microvascular thrombosis during severe infection has been also considered [85]. To date, data are scarce to support either one or the other strategy. Moreover, heparin exhibits non-anticoagulant effects as abilities to bind to inflammatory cytokines, to inhibit neutrophil chemotaxis and leukocyte migration through the endothelium, to neutralize the positively charged peptide complement factor C5a, and to sequester acute phase proteins [87, 88]. Thus, heparin anti-inflammatory functions may also be relevant in COVID-19 patients. Furthermore, Wang et al. have also reported the use of fibrinolytic agent, tissue plasminogen activator, in 3 critically ill patients, with a transient improvement of their respiratory status [89]. Eculizumab is a human monoclonal antibody designed to bind to the complement protein C5 with high affinity prevents the generation of the terminal complement complex C5b-9, which is involved in cell lysis [90]. Complement inhibition has been shown to be an effective therapeutic target in hematological and neuroinflammatory diseases [91, 92]. An Italian preliminary report of four severe COVID-19 patients treated by eculizumab recovered after treatment [93]. Currently, two French studies are testing eculizumab in SARS-CoV-2 infection, hypothesizing that complement activation may be a key player in COVID-19 infection-related EC dysfunction and multi-organ failure (Clinical Trials NCT04346797 and NCT04355494).

## Conclusion

Emerging data suggest a crucial role of endothelial dysfunction during SARS-CoV-2 infection, as a direct target of the virus and inflammatory cytokines as well as the main actor in orchestrating a pro-inflammatory and pro-coagulant state in COVID-19 patients. Promising therapies that impact endothelial dysfunction are currently under evaluation.

## Data Availability

Not applicable

## Abbreviations

ACE2: Angiotensin-converting enzyme 2
Ag: Antigen
ARDS: Acute respiratory distress syndrome
CRS: Cytokine release syndrome
DIC: Disseminated intravascular coagulation
ECs: Endothelial cells
FDP: Fibrin degradation product
ICU: Intensive care unit
IL-1β: Interleukin 1β
IL-2R: Interleukin 2 receptor
IL-6: Interleukin 6
IL-6R: Interleukin 6 receptor
ISTH: International Society on Thrombosis and Haemostasis
MCP-1: Monocyte chemoattractant
MERS-CoV: Middle East respiratory syndrome coronavirus
PAI 1: Plasminogen activator inhibitor 1
rACE2: Recombinant angiotensin-converting enzyme 2
SARS-CoV: Severe acute respiratory syndrome coronavirus
SARS-Cov-2: Severe acute respiratory syndrome coronavirus 2
TMPRSS-2: Transmembrane protease serine 2
TNF- α: Tumor necrosis factor-α
vWF: von Willebrand factor
vWF Ag: von Willebrand factor antigen
